# Risk factors for progression of supraspinatus tear size and fatty infiltration in nonsurgically treated rotator cuff tears

**DOI:** 10.1016/j.xrrt.2026.100683

**Published:** 2026-02-05

**Authors:** Yoshihiro Nakamura, Shin Yokoya, Yohei Harada, Chikara Watanabe, Shuhei Matsumura, Takahiko Hamasaki, Eisaku Fujimoto, Nobuo Adachi

**Affiliations:** a Department of Orthopaedic Surgery, Chugoku Rosai Hospital, Hiroshima, Japan; b Department of Orthopaedic Surgery, Hiroshima City Hiroshima Citizens Hospital, Hiroshima, Japan; c Department of Orthopaedic Surgery, Hiroshima University, Hiroshima, Japan

**Keywords:** Rotator cuff tear, Supraspinatus, Subscapularis, Conservative treatment, Nonsurgical treatment, Tear progression, Fatty infiltration, Risk factor

## Abstract

**Background:**

There is no clear information on structural changes of rotator cuff tears during nonsurgical management. In particular, the risk factors for the progression of tear size and fatty infiltration still remain poorly understood. Identifying such factors is essential for optimizing treatment strategies and follow-up planning.

**Methods:**

We retrospectively examined 80 shoulders (43 men, 37 women; mean age, 68.4 years) with partial- and full-thickness rotator cuff tears treated nonsurgically. All patients underwent initial and follow-up magnetic resonance imaging examinations at ≥12 months, with a mean follow-up of 22.2 ± 12.6 months. Progression of supraspinatus (SSP) tear size was defined as a mediolateral increase of ≥10 mm or ≥5 mm/yr, and progression of SSP fatty infiltration was defined as an increase in the modified Goutallier classification. Univariate analyses were conducted to determine potential risk factors, including age, sex, initial tear size, subscapularis (SSC) tear, infraspinatus (ISP) tear, initial fatty infiltration of SSC, SSP, and ISP, critical shoulder angle (CSA), and the presence of persistent pain at follow-up. Factors with significant associations were entered into multivariate logistic regression for determining independent predictors.

**Results:**

On follow-up magnetic resonance imaging, both SSP tear size and fatty infiltration demonstrated significant progression. Univariate analyses revealed a significant association between tear size progression and initial tear size (*P* < .001), SSC fatty infiltration (*P* = .004), and larger CSA (*P* = .013). SSP fatty infiltration progression was significantly associated with older age (*P* = .04), initial tear size (*P* = .01), SSC tear (*P* = .03), SSC fatty infiltration (*P* < .001), and larger CSA (*P* = .04). Multivariate logistic regression identified medium-sized tears (odds ratio [OR], 17.1; 95% confidence interval [CI], 4.3-68.3; *P* < .001) and SSC fatty infiltration grade ≥ 2 (OR, 8.1; 95% CI, 1.7-38.5; *P* = .009) as independent risk factors for SSP tear size progression, whereas CSA was not significant (OR, 1.1; *P* = .2). For fatty infiltration progression, SSC fatty infiltration grade ≥ 2 was an independent risk factor (OR, 10.4; 95% CI, 1.7-64.1; *P* = .01), whereas age (OR, 1.2; *P* = .05), medium-sized tears (OR, 3.3; *P* = .13), SSC complete tear (OR, 1.9; *P* = .5), and CSA (OR, 1.1; *P* = .41) were not significant.

**Conclusion:**

Progression of SSP tear size and fatty infiltration during nonsurgical treatment is closely associated with SSC fatty infiltration. Monitoring of SSC involvement may help identify patients at higher risk for progression of SSP tear size and fatty infiltration and support decisions.

## Introduction

A rotator cuff tear is among the most common causes of shoulder pain and functional limitation in adults. However, the optimal treatment approach remains controversial, because some tears are asymptomatic and several symptomatic cases demonstrate improvement in pain and range of motion with conservative management.^14^^,^^17^^,^^21^^,^^35^ Moreover, surgical indications are not standardized, and treatment decisions often vary among surgeons.^6^^,^^34^ Conservative treatment is frequently chosen as the initial approach because a substantial proportion of patients achieve symptomatic relief and functional improvement, although reported success rates vary widely across studies.^14^^,^^16^^,^^17^^,^^21^ Surgical repair may be undertaken when conservative management fails to provide adequate improvement. Large or massive tears are strongly associated with poorer surgical outcomes and higher rates of repair failure.^3^^,^^13^^,^^30^ Furthermore, structural changes within the rotator cuff muscles, such as atrophy and fatty infiltration, are well-recognized prognostic factors that influence post-operative outcomes.^3^^,^^10^ When either the tear size or fatty infiltration progresses considerably during nonsurgical management, the tear may become irreparable by the time surgical intervention is undertaken. Despite the clinical significance of this issue, there is a lack of clear understanding of the natural course of tear enlargement and fatty infiltration in conservatively managed rotator cuff tears. Therefore, it is essential to identify the risk factors for progression to guide treatment decisions and optimize patient outcomes. Accordingly, this study was conducted to explore the risk factors for the progression of tear size and fatty infiltration in patients with rotator cuff tears who received nonsurgical management, specifically focusing on the supraspinatus (SSP) tendon, the most commonly torn tendon of the rotator cuff.

## Materials and methods

### Study population

This study was approved by the institutional ethics committee (EPI-734). We retrospectively reviewed the data of patients diagnosed on magnetic resonance imaging [MRI] with partial- or full-thickness SSP tendon tears between 2010 and 2014. Shoulders with SSP fatty infiltration grade 4 on the modified Goutallier classification at the initial MRI examination were excluded due to end-staged degeneration. All patients received nonsurgical management consisting of anti-inflammatory analgesics, steroid injections, hyaluronic acid injections, or a combination of these treatments. Surgical repair was recommended when symptoms persisted after at least 3 months of nonsurgical care, and patients who declined surgery continued nonsurgical care and were included. Nonsurgical management was discontinued when the patients became asymptomatic, meaning the absence of pain and no clinically relevant limitation in shoulder range of motion, after which they were placed under regular follow-up. Follow-up MRI was required at ≥12 months after the initial scan. Exclusion criteria consisted of previous surgery on the affected shoulder, proximal humeral fracture, osteoarthritis, septic arthritis, rheumatoid arthritis, cervical spondylotic amyotrophy, and neuromuscular disease. Finally, 80 shoulders of 80 patients (43 men, 37 women; mean age, 68.4 ± 8.2 years) with a mean follow-up of 22.2 ± 12.6 (range, 12-50) months were enrolled.

### Magnetic resonance imaging acquisition

All MRI examinations were conducted on a 1.5-T system (Signa, GE Medical Systems, or Excelart Vantage, Toshiba). Patients underwent MRI in the supine position with the arm at the side and the palm facing upward. The protocol included oblique coronal T2-weighted, axial T2-weighted, and oblique sagittal T1- and T2-weighted sequences. The same scanner was used for the initial and follow-up examinations in each patient.

### Evaluation

Several imaging parameters were examined on both initial and follow-up MRI examinations, and changes between time points were recorded. Tear size of the SSP tendon was measured on oblique coronal T2-weighted images as the mediolateral retraction distance from the lateral edge of the greater tuberosity to the torn tendon edge, and this method was applied to both partial- and full-thickness tears. Tear size of the SSP tendon was then classified into the following 4 categories: partial-thickness, small full-thickness (≤1 cm), medium full-thickness (> 1 to ≤3 cm), and large/massive full-thickness (>3 cm).^4^ Subscapularis (SSC) tendon tears were diagnosed using axial T2-weighted images. A partial tear was defined based on high signal intensity in the superior portion of the tendon without full-thickness involvement, whereas a complete tear was defined as detachment at the insertion site. Infraspinatus (ISP) tendon tears were identified on oblique sagittal T2-weighted images when a full-thickness tear involved any portion of the middle facet. Fatty infiltration of the SSC, SSP and ISP was evaluated using the modified Goutallier classification on oblique sagittal T1-weighted images, using the most lateral slice contacting both the scapular spine and body.^8^^,^^11^ The critical shoulder angle (CSA) was measured on the initial standardized true anteroposterior radiographs of the shoulder.^26^ Moreover, the presence of shoulder pain at the time of follow-up MRI was recorded from clinical documentation. Tear size progression of the SSP tendon was defined on follow-up MRI as either (1) an absolute increase of ≥10 mm compared with the initial MRI or (2) an increase of ≥5 mm/yr of follow-up; meeting either criterion was considered tear progression. This definition was used to minimize the influence of differences in follow-up duration among patients. Fatty infiltration progression of the SSP was defined as an increase in the modified Goutallier classification grade at follow-up.

### Statistical analysis

Comparisons of SSP tear size and fatty infiltration between initial and follow-up MRI examinations were conducted using the Wilcoxon signed-rank test. The Mann–Whitney *U* test was used to evaluate differences in age, follow-up period, and CSA between progression and nonprogression cases for the progression of tear size and fatty infiltration, respectively. Fisher exact test was performed for evaluating categorical variables, including sex, initial tear size category, SSC and ISP tear status, initial fatty infiltration of the SSC, SSP, and ISP, and the presence of persistent pain at follow-up, between progression and nonprogression cases for the progression of both tear size and fatty infiltration. *P* < .05 was considered statistically significant. Variables with significant differences in univariate analyses were entered into separate multivariate logistic regression models for identifying independent predictors of progression of tear size and SSP fatty infiltration. Odds ratios (ORs) with 95% confidence intervals (CIs) were calculated. All statistical analyses were performed using EZR (Easy R; Saitama Medical Center, Jichi Medical University, Saitama, Japan), which serves as a graphical user interface for R (The R Foundation for Statistical Computing, Vienna, Austria).

## Results

The SSP tear size increased from 18.2 ± 12.1 mm at the initial MRI to 23.0 ± 13.6 mm on the follow-up MRI (*P* < .001), with a mean progression of 4.8 ± 6.2 mm, corresponding to 3.3 ± 5.5 mm/yr. The SSP fatty infiltration grade progressed from 1.8 ± 0.6 at the initial MRI to 2.0 ± 0.8 on the follow-up MRI (*P* < .001).

### Progression of SSP tear size

In the univariate comparisons between SSP tear size progression and nonprogression cases, significant differences in the initial tear size category and SSC fatty infiltration were observed between groups. The CSA was significantly larger in the progression cases. Age, sex, follow-up period, SSC tear status, ISP tear, SSP fatty infiltration, ISP fatty infiltration, or the presence of persistent pain at follow-up showed no significant differences (Table I). On the basis of these results, the following 3 factors were included in the multivariate model: medium-sized tear, SSC fatty infiltration grade ≥ 2, and CSA. In the post hoc pairwise Fisher exact test, medium-sized tears showed *P* values of <.1 compared with all other tear size categories, and SSC fatty infiltration grades ≥ 2 showed *P* values of <.1 compared with grades 0-1. Hence, medium-sized tear and SSC fatty infiltration grade ≥ 2 were selected as representative variables for the multivariate logistic regression analysis, which identified medium-sized tears (OR, 17.1; 95% CI, 4.3-68.3; *P* < .001) and SSC fatty infiltration grade ≥ 2 (OR, 8.1; 95% CI, 1.7-38.5; *P* = .009) as independent risk factors for tear progression, whereas CSA was not significant (Table II).

**Table I:** Comparison between supraspinatus tear size progression and nonprogression cases.

	Tear size progression (n = 24)	Tear size nonprogression (n = 56)	*P* value
Age (yr)	70.8 ± 5.0	67.3 ± 9.1	.19
Sex			.46
Male	45.8%	57.1%	
Female	54.2%	42.9%	
Follow-up period (mo)	21.7 ± 12.3	23.3 ± 13.6	.85
Initial tear size category			**<.001**
Partial	0%	46.4%	
Small	4.2%	8.9%	
Medium	79.2%	21.4%	
Large/massive	16.7%	23.2%	
Initial SSC tear			.13
Intact	41.7%	62.5%	
Partial	29.2%	25.0%	
Complete	29.2%	12.5%	
Initial ISP tear	20.8%	14.3%	.52
Initial fatty infiltration SSC			**.004**
0-1	58.3%	89.3%	
2	25.0%	7.1%	
3-4	16.7%	3.6%	
Initial fatty infiltration SSP			.35
0-1	16.7%	32.1%	
2	75.0%	58.9%	
3	8.3%	8.9%	
Initial fatty infiltration ISP			.17
0-1	75.0%	89.3%	
2	16.7%	8.9%	
3-4	8.3%	1.8%	
CSA (°)	36.8 ± 3.7	34.6 ± 4.1	**.013**
Pain at follow-up	62.5%	53.6%	.62

*CSA*, critical shoulder angle; *ISP*, infraspinatus; *SSC*, subscapularis; *SSP*, supraspinatus.

Bold values indicate statistical significance (*P* < .05).

**Table II:** Multivariate logistic regression analysis of risk factors associated with tear size progression.

	OR	95% CI	*P* value
Medium-sized tear	17.1	4.3-68.3	**<.001**
SSC fatty infiltration ≥ grade 2	8.1	1.7-38.5	**.009**
CSA	1.1	1.0-1.3	.20

*CI*, confidence interval; *CSA*, critical shoulder angle; *OR*, odds ratio; *SSC*, subscapularis.

Bold values indicate statistical significance (*P* < .05).

### Progression of SSP fatty infiltration

In the univariate comparisons of SSP fatty infiltration between progression and nonprogression cases, the initial tear size category, SSC tear, and SSC fatty infiltration exhibited significant differences between groups. Age was significantly higher and CSA was also significantly larger in progression cases. Sex, follow-up period, ISP tear, SSP fatty infiltration, ISP fatty infiltration, or the presence of persistent pain at follow-up showed no significant differences (Table III). Based on these findings, the following 5 factors were entered into the multivariate model: age, medium-sized tear, SSC complete tear, SSC fatty infiltration grade ≥ 2, and CSA. In the post hoc pairwise Fisher exact test, medium-sized tears compared with partial-thickness tears, SSC complete tears compared with intact SSC, and SSC fatty infiltration grade ≥ 2 compared with grades 0-1 all showed *P* values of <.1. Hence, we selected medium-sized tear, SSC complete tear, and SSC fatty infiltration grade ≥ 2 as representative variables for the multivariate logistic regression analysis, which identified SSC fatty infiltration grade ≥ 2 (OR, 10.4; 95% CI, 1.7-64.1; *P* = .01) as an independent risk factor for the progression of SSP fatty infiltration, whereas age, medium-sized tear, SSC complete tear, and CSA were not significant (Table IV).

**Table III:** Comparison between supraspinatus fatty infiltration progression and nonprogression cases.

	Fatty infiltration progression (n = 14)	Fatty infiltration nonprogression (n = 66)	*P* value
Age (yr)	72.4 ± 4.6	67.5 ± 8.6	**.04**
Sex			.15
Male	35.7%	57.6%	
Female	64.3%	42.4%	
Follow-up period (mo)	21.7 ± 13.4	24.6 ± 7.9	.05
Initial tear size category			**.01**
Partial	0%	39.4%	
Small	7.1%	7.6%	
Medium	64.3%	33.3%	
Large/massive	28.6%	19.7%	
Initial SSC tear			**.03**
Intact	35.7%	60.6%	
Partial	21.4%	27.3%	
Complete	42.9%	12.1%	
Initial ISP tear	21.4%	15.2%	.69
Initial fatty infiltration SSC			**<.001**
0-1	42.9%	87.9%	
2	28.6%	9.1%	
3-4	28.6%	3.0%	
Initial fatty infiltration SSP			.48
0-1	14.3%	30.3%	
2	78.6%	60.6%	
3	7.1%	9.1%	
Initial fatty infiltration ISP			.51
0-1	78.6%	86.4%	
2	14.3%	10.6%	
3-4	7.1%	3.0%	
CSA (°)	37.4 ± 4.7	34.8 ± 3.8	**.04**
Pain at follow-up	57.1%	54.5%	.76

*CSA*, critical shoulder angle; *ISP*, infraspinatus; *SSC*, subscapularis; *SSP*, supraspinatus.

Bold values indicate statistical significance (*P* < .05).

**Table IV:** Multivariate logistic regression analysis of risk factors associated with fatty infiltration progression.

	OR	95% CI	*P* value
Age	1.2	1.0-1.4	.05
Medium-sized tear	3.3	0.7-15.3	.13
SSC complete tear	1.9	0.3-12.8	.50
SSC fatty infiltration ≥ grade 2	10.4	1.7-64.1	**.01**
CSA	1.1	0.9-1.3	.41

*CI*, confidence interval; *CSA*, critical shoulder angle; *OR*, odds ratio; *SSC*, subscapularis.

Bold values indicate statistical significance (*P* < .05).

## Discussion

This study explored the risk factors for the progression of SSP tear size and fatty infiltration during nonsurgical management. Multivariate logistic regression analysis revealed medium-sized tears and SSC fatty infiltration as independent predictors of SSP tear progression, whereas SSC fatty infiltration was identified as the only independent predictor of SSP fatty infiltration progression.

Previous studies have identified various factors associated with rotator cuff tear progression. Patient-related factors reported to be associated with tear progression include older age,^24^ persistent pain at follow-up,^29^ smoking,^36^ diabetes,^15^ and heavy physical work.^19^ Radiologic factors associated with progression include full-thickness tears,^18^^,^^24^^,^^36^ medium-sized tears,^28^^,^^36^ and fatty infiltration or muscle atrophy of the rotator cuff.^15^^,^^19^^,^^24^ Several studies have consistently shown that full-thickness tears are more likely to progress, and medium-sized tears have also frequently been identified as a risk factor. In contrast, small or partial-thickness SSP tears have been reported to show limited progression.^7^^,^^20^^,^^32^ Overall, these findings highlight the heterogeneity of reported risk factors and definitions of tear progression, and a clear consensus has not yet been established.

CSA reflects glenoid inclination and lateral acromial extension and has been associated with rotator cuff pathology.^9^^,^^26^ Several studies have explored the relationship between CSA and tear progression, with inconsistent results, including reports showing no significant association.^2^^,^^19^ In both the single previous study and the present study, CSA showed a significant association with tear progression in univariate analysis, but this association was not maintained in multivariate analysis.^15^ Previous studies have indicated that larger rotator cuff tear sizes are associated with greater CSA values.^1^^,^^33^ Overall, these findings suggest that partial-thickness and small tears, which are less likely to progress, tend to be associated with smaller CSA values, and that tear type and tear size could have acted as confounding factors.

In the present study, SSC fatty infiltration grade ≥ 2 was identified as an independent risk factor for SSP tear enlargement. A few previous studies have investigated the relationship between SSC fatty infiltration and tear progression, reporting no significant association, probably because SSC complete tears were scarcely included in their cohorts.^15^^,^^19^ In contrast, our study included cases with SSC complete tears and thus provides a novel finding. We speculate that this could be related to the “comma sign.” The comma sign is an arthroscopic landmark for identifying and repairing SSC tears. It was first described by Lo and Burkhart^23^ in 2003 and represents an arc formed by a portion of the superior glenohumeral ligament and coracohumeral ligament complex, serving as a useful marker of the superolateral corner of the torn SSC tendon. In rotator cuff repair, when an SSC tear is continuous with an SSP tear, repairing the SSC first allows the anterior portion of the posterosuperior cuff to be mobilized laterally with reduced tension, thereby facilitating a more secure repair.^5^^,^^12^^,^^31^ This effect is mediated through reduction of the comma tissue, which also contributes to SSP tendon reduction through its connection to the anterior rotator cable. In contrast, in rotator cuff tears that are treated nonsurgically, the opposite mechanism may occur, ie, when the SSC tear advances, the SSP tendon can be retracted medially together with the SSC tendon through the comma tissue. This could explain why SSC fatty infiltration was identified as a risk factor for SSP tear enlargement in our study.

In symptomatic rotator cuff tears that receive nonsurgical treatment, several studies have reported that muscle atrophy and fatty infiltration of the rotator cuff tend to progress over time.^7^^,^^15^^,^^19^^,^^24^^,^^27^ Nevertheless, there are scarce investigations into their specific risk factors. One study reported that massive tears are more likely to exhibit progression of muscle atrophy,^25^ but no clear predictors of muscle atrophy and fatty infiltration progression have been established. In the present study, we identified SSC fatty infiltration as a risk factor for the progression of SSP fatty infiltration. The mechanism underlying this finding may also be explained by the “comma sign” theory. In SSC tears with advanced fatty infiltration, the continuity of the anterior portion of the SSP is compromised at the comma tissue, which may predispose the SSP to further fatty infiltration. Overall, these findings suggest that patients with progressive SSC fatty infiltration need particularly careful follow-up during nonsurgical management of rotator cuff tears to decide on treatment strategies before the SSP becomes irreparable.

This study has several limitations. First, this retrospective study is subject to selection bias, as patients who proceeded to surgery after persistent symptoms were excluded, resulting in a cohort limited to those continuing nonsurgical management. Second, the details of nonsurgical management varied among patients and were not standardized. Third, detailed patient-related factors such as dominant hand, occupational status, smoking, and diabetes mellitus were not consistently available due to the retrospective nature of this study. Fourth, the intervals between initial and follow-up MRI varied, preventing an accurate determination of the timing of progression. Absolute changes were affected by the follow-up duration, whereas annualized changes were unreliable because tear enlargement is not constant. Consequently, tear size progression was defined using both absolute and annual criteria. Fifth, fatty infiltration was evaluated qualitatively using the modified Goutallier classification, which has limited reproducibility and may have introduced bias.^22^

## Conclusion

In symptomatic rotator cuff tears that are treated nonsurgically, medium-sized tears and SSC fatty infiltration grade ≥ 2 were identified as independent predictors of SSP tear size progression, and SSC fatty infiltration grade ≥ 2 also predicted SSP fatty infiltration progression. Therefore, careful monitoring of SSC fatty infiltration is essential in patients receiving nonsurgical management.

## Disclaimers:

Funding: No external funding was received for this study.

Conflicts of interest: The authors, their immediate families, and any research foundations with which they are affiliated have not received any financial payments or other benefits from any commercial entity related to the subject of this article.

## References

[1] Blonna D., Giani A., Bellato E., Mattei L., Caló M., Rossi R. (2016). Predominance of the critical shoulder angle in the pathogenesis of degenerative diseases of the shoulder. J Shoulder Elbow Surg.

[2] Chalmers P.N., Salazar D., Steger-May K., Chamberlain A.M., Yamaguchi K., Keener J.D. (2017). Does the critical shoulder angle correlate with rotator cuff tear progression?. Clin Orthop Relat Res.

[3] Cho N.S., Lee B.G., Rhee Y.G. (2011). Arthroscopic rotator cuff repair using a suture bridge technique: is the repair integrity actually maintained?. Am J Sports Med.

[4] DeOrio J.K., Cofield R.H. (1984). Results of a second attempt at surgical repair of a failed initial rotator-cuff repair. J Bone Joint Surg Am.

[5] Dilisio M.F., Neyton L. (2014). Comma sign-directed repair of anterosuperior rotator cuff tears. Arthrosc Tech.

[6] Dunn W.R., Schackman B.R., Walsh C., Lyman S., Jones E.C., Warren R.F. (2005). Variation in orthopaedic surgeons' perceptions about the indications for rotator cuff surgery. J Bone Joint Surg Am.

[7] Fucentese S.F., von Roll A.L., Pfirrmann C.W., Gerber C., Jost B. (2012). Evolution of nonoperatively treated symptomatic isolated full-thickness supraspinatus tears. J Bone Joint Surg Am.

[8] Fuchs B., Weishaupt D., Zanetti M., Hodler J., Gerber C. (1999). Fatty degeneration of the muscles of the rotator cuff: assessment by computed tomography versus magnetic resonance imaging. J Shoulder Elbow Surg.

[9] Gerber C., Snedeker J.G., Baumgartner D., Viehöfer A.F. (2014). Supraspinatus tendon load during abduction is dependent on the size of the critical shoulder angle: a biomechanical analysis. J Orthop Res.

[10] Gladstone J.N., Bishop J.Y., Lo I.K., Flatow E.L. (2007). Fatty infiltration and atrophy of the rotator cuff do not improve after rotator cuff repair and correlate with poor functional outcome. Am J Sports Med.

[11] Goutallier D., Postel J.M., Bernageau J., Lavau L., Voisin M.C. (1994). Fatty muscle degeneration in cuff ruptures. Pre- and postoperative evaluation by CT scan. Clin Orthop Relat Res.

[12] Gröger F., Hackl M., Buess E. (2022). Arthroscopic suture-bridge repair of the Subscapularis Tendon-"Inside and outside the box" with preservation of the comma sign. Arthrosc Tech.

[13] Gulotta L.V., Nho S.J., Dodson C.C., Adler R.S., Altchek D.W., MacGillivray J.D. (2011). Prospective evaluation of arthroscopic rotator cuff repairs at 5 years: part II--prognostic factors for clinical and radiographic outcomes. J Shoulder Elbow Surg.

[14] Itoi E., Tabata S. (1992). Conservative treatment of rotator cuff tears. Clin Orthop Relat Res.

[15] Jung W., Lee S., Hoon Kim S. (2020). The natural course of and risk factors for tear progression in conservatively treated full-thickness rotator cuff tears. J Shoulder Elbow Surg.

[16] Kane L.T., Luthringer T., Vaughan A., Kim S., Ramsey M.L., Namdari S. (2024). Outcomes of initial nonoperative treatment of traumatic full-thickness rotator cuff tears. J Shoulder Elbow Surg.

[17] Kijima H., Minagawa H., Nishi T., Kikuchi K., Shimada Y. (2012). Long-term follow-up of cases of rotator cuff tear treated conservatively. J Shoulder Elbow Surg.

[18] Kim Y.S., Kim S.E., Bae S.H., Lee H.J., Jee W.H., Park C.K. (2017). Tear progression of symptomatic full-thickness and partial-thickness rotator cuff tears as measured by repeated MRI. Knee Surg Sports Traumatol Arthrosc.

[19] Ko S.H., Na S.C., Kim M.S. (2023). Risk factors of tear progression in symptomatic small to medium-sized full-thickness rotator cuff tear: relationship between occupation ratio of supraspinatus and work level. J Shoulder Elbow Surg.

[20] Kong B.Y., Cho M., Lee H.R., Choi Y.E., Kim S.H. (2018). Structural evolution of nonoperatively treated high-grade partial-thickness tears of the supraspinatus tendon. Am J Sports Med.

[21] Kuhn J.E., Dunn W.R., Sanders R., An Q., Baumgarten K.M., Bishop J.Y. (2013). Effectiveness of physical therapy in treating atraumatic full-thickness rotator cuff tears: a multicenter prospective cohort study. J Shoulder Elbow Surg.

[22] Lippe J., Spang J.T., Leger R.R., Arciero R.A., Mazzocca A.D., Shea K.P. (2012). Inter-rater agreement of the Goutallier, Patte, and Warner classification scores using preoperative magnetic resonance imaging in patients with rotator cuff tears. Arthroscopy.

[23] Lo I.K., Burkhart S.S. (2003). The comma sign: an arthroscopic guide to the torn subscapularis tendon. Arthroscopy.

[24] Maman E., Harris C., White L., Tomlinson G., Shashank M., Boynton E. (2009). Outcome of nonoperative treatment of symptomatic rotator cuff tears monitored by magnetic resonance imaging. J Bone Joint Surg Am.

[25] Matsumura N., Kiyota Y., Suzuki T., Iwamoto T., Nozaki T., Jinzaki M. (2024). Quantitative evaluation of natural progression of fatty infiltration and muscle atrophy in chronic rotator cuff tears without tear extension using magnetic resonance imaging. JSES Int.

[26] Moor B.K., Bouaicha S., Rothenfluh D.A., Sukthankar A., Gerber C. (2013). Is there an association between the individual anatomy of the scapula and the development of rotator cuff tears or osteoarthritis of the glenohumeral joint?: a radiological study of the critical shoulder angle. Bone Joint J.

[27] Nakamura Y., Yokoya S., Harada Y., Shiraishi K., Adachi N., Ochi M. (2017). The prospective evaluation of changes in fatty infiltration and shoulder strength in nonsurgically treated rotator cuff tears. J Orthop Sci.

[28] Nakamura Y., Yokoya S., Mochizuki Y., Harada Y., Kikugawa K., Ochi M. (2015). Monitoring of progression of nonsurgically treated rotator cuff tears by magnetic resonance imaging. J Orthop Sci.

[29] Safran O., Schroeder J., Bloom R., Weil Y., Milgrom C. (2011). Natural history of nonoperatively treated symptomatic rotator cuff tears in patients 60 years old or younger. Am J Sports Med.

[30] Sugaya H., Maeda K., Matsuki K., Moriishi J. (2007). Repair integrity and functional outcome after arthroscopic double-row rotator cuff repair. A prospective outcome study. J Bone Joint Surg Am.

[31] Ticker J.B., Burkhart S.S. (2011). Why repair the subscapularis? A logical rationale. Arthroscopy.

[32] Tsuchiya S., Davison E.M., Rashid M.S., Bois A.J., LeBlanc J., More K.D. (2021). Determining the rate of full-thickness progression in partial-thickness rotator cuff tears: a systematic review. J Shoulder Elbow Surg.

[33] Tunalı O., Erşen A., Kızılkurt T., Bayram S., Sıvacıoğlu S., Atalar A.C. (2022). Are critical shoulder angle and acromion index correlated to the size of a rotator cuff tear. Orthop Traumatol Surg Res.

[34] Wolf B.R., Dunn W.R., Wright R.W. (2007). Indications for repair of full-thickness rotator cuff tears. Am J Sports Med.

[35] Yamamoto A., Takagishi K., Kobayashi T., Shitara H., Osawa T. (2011). Factors involved in the presence of symptoms associated with rotator cuff tears: a comparison of asymptomatic and symptomatic rotator cuff tears in the general population. J Shoulder Elbow Surg.

[36] Yamamoto N., Mineta M., Kawakami J., Sano H., Itoi E. (2017). Risk factors for tear progression in symptomatic rotator cuff tears: a prospective study of 174 shoulders. Am J Sports Med.

